# Identification and Expression of the *MADS-box* Gene Family in Different Versions of the *Ginkgo biloba* Genome

**DOI:** 10.3390/plants12183334

**Published:** 2023-09-21

**Authors:** Pengyan Zhou, Zesen Wang, Yingang Li, Qi Zhou

**Affiliations:** 1 Zhejiang Academy of Forestry, 399 Liuhe Road, Hangzhou 310023, China; pyzhou@njfu.edu.cn (P.Z.); hzliyg@126.com (Y.L.); 2 Co-Innovation Center for Sustainable Forestry in Southern China, Nanjing Forestry University, Nanjing 210037, China; wzs158159@163.com

**Keywords:** Ginkgo genome, *MADS-box*, gene structure, expression

## Abstract

*MADS-box* transcription factors play important roles in many organisms. These transcription factors are involved in processes such as the formation of the flower organ structure and the seed development of plants. *Ginkgo biloba* has two genome versions (version 2019 and version 2021), and there is no analysis or comparison of the *MADS-box* gene family in these two genomes. In this study, 26 and 20 *MADS-box* genes were identified from the two genomes of Ginkgo, of which 12 pairs of genes reached more than 80% similarity. According to our phylogenetic analysis results, we divided these genes into type I (Mα and Mγ subfamilies) and type II (MIKC and Mδ subfamilies) members. We found that both sets of genomes lacked the Mβ gene, while the MIKC gene was the most numerous. Further analysis of the gene structure showed that the MIKC genes in the two genomes had extralong introns (≥20 kb); these introns had different splicing patterns, and their expression might be more abundant. The gene expression analysis proved that *GbMADS* genes were expressed to varying degrees in eight Ginkgo biological tissues. Type II *GbMADS* genes not only were found to be related to female flower bud differentiation and development but also are important in seed development. Therefore, *MADS-box* genes may play important roles in the development of Ginkgo reproductive organs, which may suggest a genetic role in sexual differentiation. This study further contributes to the research on *MADS-box* genes and provides new insights into sex determination in Ginkgo.

## 1. Introduction

*MADS-box* gene family members encode transcription factors (TFs), which play an important role in many biological functions in eukaryotes [1]. *MADS-box* TFs are characterized by the presence of a DNA-binding domain of approximately 60 amino acids (aa) in length, which is collectively called the MADS domain. This domain was located in the N-terminal region of the protein. One of the most notable features of *MADS-box* gene family members is their important role in the flowering ABCDE model of plants [2]. Researchers have conducted many studies in *Arabidopsis thaliana*, *Oryza sativa* L., *Populus* L., etc. [3,4,5]. The *MADS-box* gene has been shown to be critical in regulating plant development, for example, in female gametophytes, seed development, and fruit ripening [6]. It was reported that *MADS-box* genes also participate in responses to stress, both abiotic and biotic stress [7]. For example, the expression of *TaMADS2* is upregulated after wheat stripe rust infection [8], and some *MADS-box* genes might also be involved in responses to high salt concentrations [9]. In addition, recent progress that has been made regarding the roles of *MADS* genes during gymnosperm reproduction deserves more attention. *MADS11* and *DAL1* interact to mediate the vegetative-to-reproductive transition in pine [10]. The male-specific region of the Y chromosome of Cycas contains a *MADS-box* transcription factor expressed exclusively in male cones [11]. These studies have important implications for *MADS-box* genes in gymnosperms.

Gramzow et al. classified the *MADS-box* genes into SRF-like (type I) and MEF2-like (type II) [12]. Type I included type M and type N genes [13]. In addition to the MADS domain (M), type II genes contain three additional domains, namely, an intervening domain (I), a keratin-like domain (K), and a C-terminal domain (C). Type II genes can be divided into MIKC^C^ and MIKC* genes according to their structural differences [4,14]. Bayesian classification of MADS-box proteins in *A. thaliana* clustered the proteins into five distinct groups (Mα, Mβ, Mγ, Mδ, and MIKC) [3]. This classification scheme was used in this study.

*Ginkgo biloba*, which is native to China, is the only species in the Ginkgo family [15]. Gingko fruit has antioxidant, antibacterial, and other biological functions [16]. Ginkgo is a typical hermaphroditic plant species, and there are great differences in the application of the different sexes. Female plants are mostly used for fruit production. However, due to the unpleasant smell of ripe fruit, male plants tend to be chosen for landscaping. In theory, it takes 20 to 30 years for the sex of Ginkgo seedlings to be identified according to the inflorescence, which greatly limits the promotion and application of different sexes of Ginkgo trees. Therefore, exploring the mechanism of sex differentiation in Ginkgo has become a research priority. The MADS-box gene plays an important role in flower bud differentiation, while its role in Ginkgo sex regulation remains unknown. Although the *MADS-box* gene family was initially identified in Ginkgo [17], the Ginkgo genome has been published in two versions. In 2019, Guan et al. updated the Ginkgo genome assembly to the chromosome level with Hi-C technology and obtained a high-quality Ginkgo genome [18]. In 2021, Liu et al. presented another Ginkgo genome assembly based on long reads (PacBio RSII platform) [19]. Considering the incomplete assembly of genomes and the possibility of annotation errors, it is particularly important to compare the differences in *MADS-box* genes between the two genomes.

Although a small number of *MADS-box* genes have also been verified in Ginkgo [20,21,22], genome-wide identification and expression analysis from two genomes is still lacking. Studies have shown that genome duplication events often lead to an increase in gene family members and redundancy in function. Ginkgo has experienced only one duplication event common to the ancestors of seed-bearing plants (~320 Mya) [19,23]. Considering the information in the iTAK database [24], the Ginkgo TF families usually have relatively few members. Therefore, studies of the function of family members tend to be representative of the species.

In this study, we comprehensively identified the *MADS-box* gene family of Ginkgo from a genome-wide perspective. For the first time, we mined MADS genes from two Ginkgo genomes simultaneously. We wanted to determine the differences in Ginkgo MADS genes in the two genomes and to analyze their structural characteristics. Through a quantitative expression analysis of the *MADS-box* gene in eight different tissues, we tried to comprehensively explore the potential function of the *MADS-box* genes in various tissues of Ginkgo. This study provides the first expression study of *MADS-box* genes within two Ginkgo genomes and has given a different idea for molecular biology research on Ginkgo.

## 2. Results

### 2.1. Identification of GbMADS Proteins

According to the results of BLAST and HMM models, 40 GbMADS candidates were initially detected throughout the version 2019 genome (v2019), and 28 candidates were initially detected throughout the version 2021 genome (v2021); their protein sequences are shown in Supplementary S1 and S2, respectively. Based on the MADS-box model in the SMART program, 26 proteins and 20 proteins with complete MADS-box domains were identified in v2019 and v2021, respectively (Table 1). In order to distinguish them, the 2019 version is named as GbMADS1-GbMADS26 and the 2021 version is named as GbMADS27-GbMADS46. Among them, there were 12 pairs of genes that reached 80% similarity. They all have a conserved MADS domain consisting of approximately 60 aa, which is located at the N-terminus. Multisequence alignment and the sequence icon of MADS domains v2019 revealed 10 highly conserved aa (17, 21, 23, 24, 27, 30, 31, 34, 35, and 39). In contrast, the MADS domains v2021 revealed 13 highly conserved aa (1, 2, 17, 21, 23, 24, 27, 30, 31, 34, 38, 39, 48) (Figure S1).

According to the alignment results of MADS domains v2019 and v2021 (Figure S2), N-terminal diversity reflected the differences between different types of MADS-box domains. In addition, type I domains were more variable than type II domains.

The results of the physicochemical properties of GbMADS proteins are shown in Table 1. The GbMADS protein v2019 was between 91 and 445 aa long, and v2021 was between 61 and 451 aa long. Most GbMADS proteins in v2019 (80.77%) and v2021 (70%) were between 100 and 400 aa long. The predicted molecular weights ranged from 10584.23 to 50951.76 kDa (v2019) and 6849.08 to 51348.86 kDa (v2021). The predicted isoelectric points ranged from 4.7 to 10.2 (v2019) and from 5.53 to 10.58 (v2021). The grand average of hydropathicity ranged from −0.754 to 0.131 (v2019) and from −0.917 to −0.159 (v2021). Subcellular localization predicted that all the proteins were localized in the nucleus.

### 2.2. Phylogenetic Analysis and Gene Structure of GbMADS

**Figure 1:**
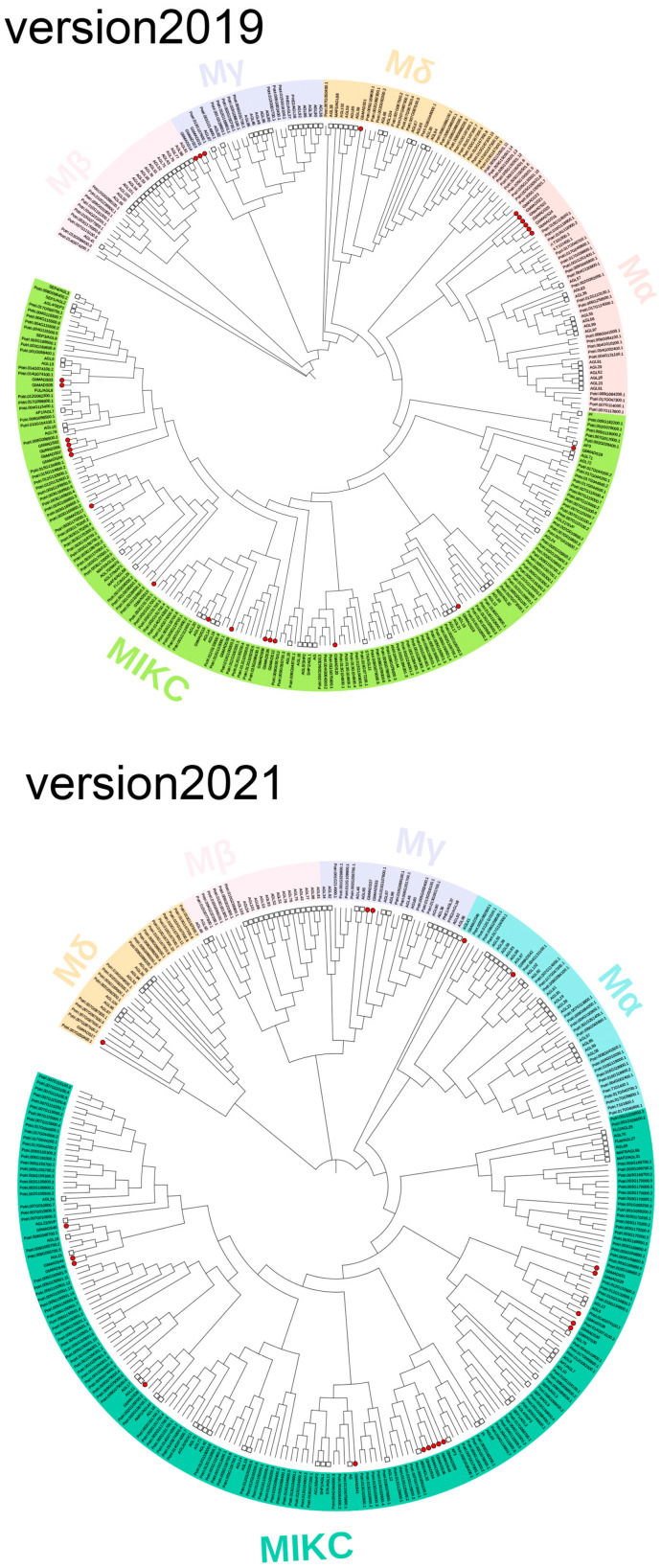
A phylogenetic tree was constructed with the ML method, which is composed of homologous MADS protein sequences in Ginkgo, *A. thaliana*, and *Populus* L. The red circle represents MADS in Ginkgo and the white square represents MADS in *A. thaliana*. Version2019 represents the MADS proteins identified from the 2019 version of the genome. Version2021 represents the MADS proteins identified from the 2021 version of the genome. For version2019, the light green represents MIKC subfamily, the dark pink represents Mα subfamily, the light pink represents Mβ subfamily, the light purple represents Mγ subfamily, and the orange represents Mδ subfamily. For version2021, the green represents MIKC subfamily, the blue represents Mα subfamily, the light pink represents Mβ subfamily, the light purple represents Mγ subfamily, and the light orange represents Mδ subfamily.

As model organisms, the classification studies of *MADS* genes in poplar and *A. thaliana* are relatively complete. Here, we refer to their classification in the *GbMADS* phylogeny study. Based on the phylogenetic trees (Figure 1), as well as the classification of genes, the *GbMADS* gene families of v2019 and v2021 could all be divided into two categories: type I (8 members in v2019 and 4 members in v2021) and type II (18 members in v2019 and 16 members in v2021). The type I genes were classified into Mα (five members in v2019 and two members in v2021) and Mγ (three members in v2019 and two members in v2021). Similarly, the type II genes could be divided into two subfamilies, MIKC (17 members in v2019 and 15 members in v2021) and Mδ (1 member in v2019 and 1 member in v2021). Overall, there were fewer GbMADS proteins than there were in *A. thaliana* and poplar. However, as in poplar and *A. thaliana*, the MIKC subfamily had the most genes. There were more type II GbMADS proteins (18 members in v2019 and 16 members in v2021) than type I GbMADS proteins (8 members in v2019 and 4 members in v2021), but the GbMADS proteins did not have members of the Mβ subfamily.

### 2.3. Gene Structure and Motif Analysis

**Figure 2:**
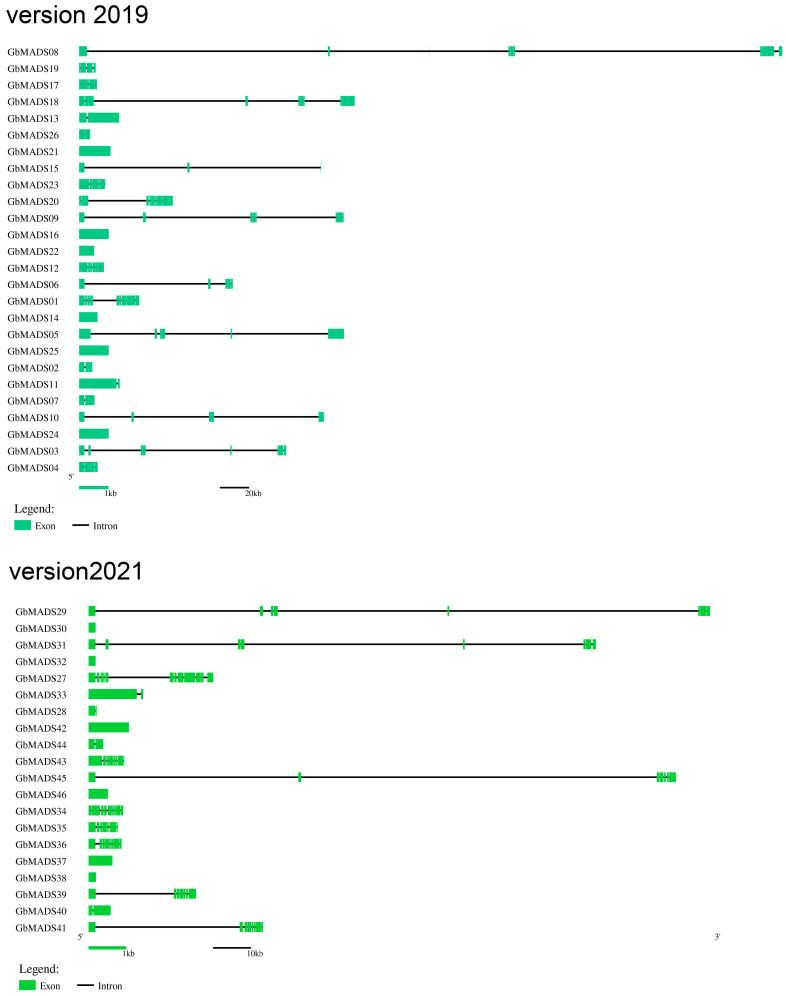
Gene structure of *GbMADS* genes. Version2019 represents the *MADS* genes identified from the 2019 version of the genome. Version2021 represents the *MADS* genes identified from the 2021 version of the genome. Green lines are exons, and black lines are introns.

The Gene Structure Display Server (GSDS) website was used for the gene structure analysis (Figure 2). For the *GbMADS* genes in genome v2019, six genes had no introns, and the remaining twenty *GbMADS* genes contained 1 to 10 introns (Table 1). With respect to type I genes, except for *GbMADS11* and *GbMADS13*, the rest did not contain introns. Type II *GbMADS* genes mostly contained two to five introns, with an average number (5.5) much greater than that in the type I *GbMADS* genes (0.5).

For the *GbMADS* genes in genome v2021, six genes had no introns, and the remaining fourteen *GbMADS* genes contained 1 to 12 introns (Table 1). Type I genes did not contain introns except for *GbMADS33*. Most type II *GbMADS* genes contained 7 to 12 introns. The average *MADS* gene number (4.25) of type II was much greater than that of type I (0.25).

Furthermore, gene introns with widely different lengths had also become our focus. In type I genes of v2019, both *GbMADS11* and *GbMADS13* had an intron length of 1 kb. Among the type II genes, 55.56% were greater than 15 kb, of which nine genes (*GbMADS03*, *05*, *06*, *08*, *09*, *10*, *15*, *18*, and *20*) had extralong introns (≥20 kb). In genome v2021, *GbMADS33* had an intron length of 1 kb. Type II genes had longer introns, and 37.5% were greater than 15 kb, of which five genes (*GbMADS29*, *31*, *39*, *41*, and *45*) had extralong introns (≥20 kb). Their introns were much longer than those of other genes.

In summary, the type II genes of both genomes have more introns than the type I genes and are longer in length. Compared with the v2019 genome, the *GbMADS* genes of v2021 have fewer introns and fewer superlong introns.

### 2.4. Interaction Network and Expression Analysis of GbMADS

**Figure 3:**
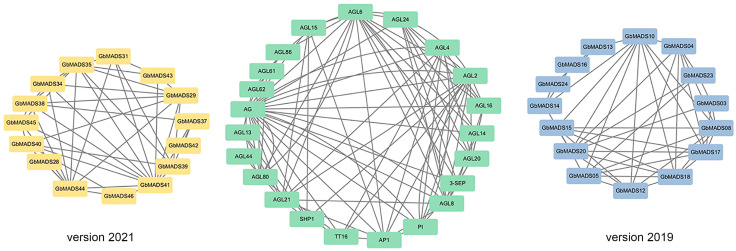
Interaction network analysis of GbMADS proteins. Version2019 represents the MADS proteins interaction in the 2019 version of the genome, and Version2021 represents the MADS proteins interaction in the 2021 version of the genome.

To determine the biological function and regulatory network of the GbMADS proteins, homologous MADS-box proteins of *A. thaliana* were used to predict the GbMADS protein–protein interaction network (Figure 3). The homology relationship between *A. thaliana* and Ginkgo MADS proteins is shown in Table S3. Based on the interaction between genes, 15 GbMADS proteins were homologous to 22 MADS proteins in *A. thaliana*. They belong to the MIKC subfamily, except for GbMADS13, 14, 16, 24, 37, 42, and 46. Most GbMADS proteins could interact with 4–12 proteins. Among the MADS proteins of *A. thaliana*, AGL6, AGL8, and AGL104 are mainly related to the development of flowers; PI, AG, AGL61, and SEP are related to the development of female and male gametophytes; and AGL62 affects early endosperm development.

**Figure 4:**
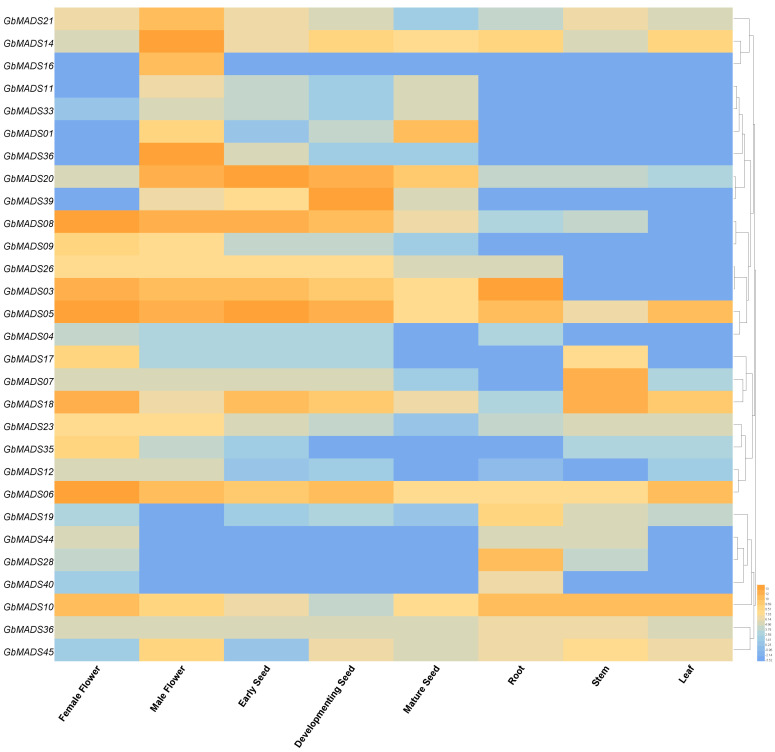
*GbMADS* gene expression analysis. Orange to blue represent expression from high to low.

Twenty-eight pairs of *GbMADS* genes were selected to explore the expression patterns in eight tissues, namely, female flowers (FF), male flowers (MF), early seeds (ES), developing seeds (DS), mature seeds (MS), roots (R), stems (S), and leaves (L) (Figure 4, Table S4). To avoid duplicate genes, we selected all genes of the 2019 version, as well as genes of the 2021 version with less than 80% similarity to the 2019 version genes (genes with greater than 80% similarity are listed in Table S2).

Overall, the most highly expressed genes were in MF and FF, followed by ES and DS. The expression of most genes was low in R, S, and L. Among type I genes, *GbMADS14* showed moderate or high expression in all tissues, with the highest expression in MF, followed by DS. *GbMADS16* and *GbMADS21* were highly expressed in MF and showed moderate or lower expression in other tissues. *GbMADS11* and *GbMADS33* showed low expression in all tissues. Among the type II genes, the Mδ gene *GbMADS01* was most highly expressed in MS, followed by MF. Among the *MIKC* genes, *GbMADS05*, *06*, and *10* were highly expressed in eight tissues. *GbMADS 03*, *20*, *26*, *08*, and *18* were highly expressed in more than half of the tissues, such as FF, MF, ES, DS, and MS. *GbMADS17*, *45*, and *39* were all highly expressed in 2~3 tissues, which were reflected in FF, MF, and DS. *GbMADS23*, *09*, *12*, *36*, *28*, *35*, *36*, *40*, *19*, and *07* were highly expressed in 1~2 tissues, while *GbMADS04* and *GbMADS44* showed low expression in all tissues. In addition, *GbMADS14* in MF, *GbMADS08* in FF, *GbMADS20* in ES, and *GbMADS39* in DS all showed particularly high expression levels. They may play key roles in plant growth and development. In summary, *GbMADS* genes, especially *MIKC* genes, may play a role in flower organ development and seed development.

## 3. Discussion

### 3.1. Number and Type of GbMADS Proteins

In the genome versions of v2019 and 2021, twenty-six and twenty members of the MADS-box gene family (GbMADS01 to GbMADS26 and GbMADS27 to GbMADS46) with complete MADS domains were finally identified, respectively. In a previous study by Yang et al., 26 genes were also identified in the v2019 genome [17]. We found that the number of GbMADS proteins was lower than that in most plant species, such as *A. thaliana* (106), poplar (105), and *Salix suchowensis* (60) [3,4,25]. The genomes of these species vary in size (*A. thaliana*, 207 Mb; poplar, 431 Mb; *S. suchowensis*, 356 Mb; Ginkgo, 9.87 Gb). However, the number of protein-coding genes in *A. thaliana* was similar to that in Ginkgo, and those of poplar and *S. suchowensis* were almost twice that in Ginkgo. The Ginkgo genome is large. The increases in genome-wide duplication and repeat sequences increased the size and complexity of the Ginkgo genome. Therefore, gene loss may have occurred frequently throughout the long evolutionary process of Ginkgo.

In reference to the classification of *A. thaliana* and poplar subfamilies for phylogenetic tree construction [3,4], type II genes accounted for a higher proportion of Ginkgo (69.23% and 80% for v2019 and v2021, respectively) than poplar (60.95%) and *Arabidopsis* (47.4%). It has been found that MIKC subfamily genes play important roles in the development of pistils and stamens of gymnosperms [26], suggesting that these genes might play the same role in the sexual differentiation of Ginkgo. Compared with *A. thaliana*, poplar, and other plant species, Ginkgo does not have Mβ genes, and similar results have been found in sesame [27]. This means that the Mβ genes do not play important roles in Ginkgo, as all of them have been lost during evolution.

### 3.2. Gene Structure of GbMADS genes

The expression of genes is related to introns. Different ways in which introns were spliced could result in different activities at different periods, resulting in different proteins. Regardless of v2019 or v2021, the type I *GbMADS* genes had a simple structure. Most of them had zero or only one intron, and they were short (<1 kb). This was similar to the homologs in *A. thaliana*. The type II genes had a higher number of introns and a more variable structure. Some MIKC genes (*GbMADS03*, *05*, *06*, *08*, *09*, *10*, *15*, *18*, *20*, *29*, *31*, *39*, *41*, and *45*) also have extralong introns (≥20 kb). Their introns were much longer than the introns of the type II *AtMADS* genes (the longest introns were only 3.8 kb). It has been suggested that the gene expression is richer in genes in which oversized introns and transposons have been inserted [28,29]. These extralong introns indicated that Ginkgo genes are relatively long, and different splicing patterns lead to the diverse expression of genes. It had been confirmed that MIKC genes had a great influence on sex determination and flower development. [26]. Ginkgo is a dioecious plant species that often takes more than 20 years to distinguish between sexes based on inflorescence characteristics. MIKC genes may have an important influence on the sex determination of Ginkgo, which is important for the early sex distinction of this species.

In addition, we found that the *GbMADS* genes of 2021 version genome had fewer gene introns and were shorter. When we compared the two genomes, we found that the scaffold N50 length of the 2019 version genome was 0.135 Mb, while the scaffold N50 length of the 2021 version genome was 775 Mb [18,19]. N50 is the evaluation index after genome assembly. The longer the length, the better the quality of the assembly. The N50 of the 2021 version is thousands of times higher than that of the 2019 version, indicating that the splicing quality of the 2021 version is better. The incompleteness of gene splicing in different versions of the genome may lead to differences in introns.

### 3.3. Gene Expression and Potential Function

Liao et al. identified nineteen *GbMADS* genes using a combination of transcriptome alignment and de novo prediction and found that the male sex-determining region of Ginkgo contained more than 200 genes. Four *GbMADS* genes that play an important role in sex differentiation were present [30]. According to studies of *A. thaliana*, *AGL80* affects central cell differentiation during female gametophyte development [31], and *AGL61* has a role in female gametophyte development [32]. In this study, we found that type I genes were specifically highly expressed in male flowers. These genes included the AGL80-like gene *GbMADS14* and the *AGL61-like* genes *GbMADS16* and *GbMADS21*. Therefore, it is thought that they may play an important role in the sex differentiation of Ginkgo.

In *A. thaliana*, *AGL2* plays a role in floral meristem tissues, thereby ensuring the normal development of petals, stamens, and carpels [33,34]. *AGL6* and *AGL16* were involved in the gene regulation and regulation of long-day flowering time during the development of peanut meristem tissue [35,36], respectively. *AGL20* affected the flowering and developmental period of *A. thaliana* [37]. Zhi Feng et al. found that the expression level of the SOC1 homolog *Gb01884* varied from low to high in the early stage of flower bud differentiation and emergence, which was based on transcriptome data from Ginkgo [38]. In this study, most of the MIKC subfamily genes were specifically highly expressed in female flowers. Therefore, the *AGL2-like* gene *GbMADS05* might have an effect on the sex determination mechanism of Ginkgo. The *AGL6-like* genes *GbMADS06* and *GbMADS09*, the *AGL16-like* gene *GbMADS17*, and the *AGL20-like* gene *GbMADS08* may regulate the differentiation of female flower buds of Ginkgo.

MIKC genes were also highly expressed in other tissues. *AGL24* (*SOC1*) is a transcriptional activator that promotes the growth of apical meristem tissue [39]. The *AGL24-like* gene *GbMADS18* exhibited a high level of expression at the ends of the stems, which might promote the growth of apical meristem tissue. In addition, we found that MIKC genes exhibited moderate or high expression in young and developing seeds. Lovisetto et al. overexpressed the *AG* homologous gene in Ginkgo and demonstrated that *AG* had a substantial role in the development of fleshy fruits in gymnosperms [40]. This is important for the evolution of seed propagation. *TT16* is involved in the developmental regulation of the endothelium and in the accumulation of proanthocyanidins or condensed tannins, which give the seed its brown pigmentation after oxidation [41]. The *AG-like* gene *GbMADS20* and *TT16-like gene GbMADS39* in this study may also play the same role in Ginkgo seeds. When the results of the analysis of the protein interaction networks and gene expression are combined, *GbMADS* genes may be considered to have some impact on the development of reproductive organs and sex determination. It may benefit from further development in the future.

## 4. Materials and Methods

### 4.1. Identification and Phylogenetic Analysis of GbMADS Proteins

The two Ginkgo genomic datasets were accessible at http://gigadb.org/dataset/100613# (accessed on 20 January 2022) and https://ginkgo.zju.edu.cn/genome/ (accessed on 21 January 2022). From the TAIR database (http://www.arabidopsis.org/) (accessed on 21 January 2022), we downloaded the MADS-box protein sequences of *A. thaliana*. Then, we aligned them to protein sequences of Ginkgo. The HMMER version 3.0 program was also applied for the Ginkgo MADS-box protein search with the Pfam accession PF00319 [42].

We verified the protein further though the SMART website (http://smart.embl-heidelberg.de/) [43] and CDD Database (https://www.ncbi.nlm.nih.gov/Structure/cdd/wrpsb.cgi) (accessed on 25 January 2022). Moreover, the MADS-box protein characteristics and subcellular location predictions were determined and carried out using ExPASy (https://web.expasy.org/protparam/) and Plant-mPLoc (http://www.csbio.sjtu.edu.cn/bioinf/plant-multi/) (accessed on 11 February 2022), respectively.

### 4.2. Sequence Alignment and Phylogenetic Analysis

The Weblogo website (http://weblogo.berkeley.edu/logo.cgi) was applied for the GbMADS logos [44]. At the same time, ClustalX version 2.1 and ESPript website (https://espript.ibcp.fr/ESPript/cgi-bin/ESPript.cgi) (accessed on 18 February 2022) were applied for sequence alignment and for coloring the results of GbMADS in the two versions of the genome.

MEGA 11.0 was used to construct the phylogenetic tree of three species: Ginkgo, populus, and *A. thaliana* [45]. MADS-box protein sequences of *Populus trichocarpa* were acquired from https://phytozome-next.jgi.doe.gov/ (accessed on 21 January 2022). The results were visualized using the iTOLs online tool (https://itol.embl.de/itol.cgi) (accessed on 25 February 2023).

### 4.3. Gene Structure and Protein–Protein Interaction Network Analysis

The exon/intron map was constructed in the GSDS online tool (http://gsds.gao-lab.org/) (accessed on 12 May 2023).

A homology analysis of MADS-box proteins in Ginkgo and *A. thaliana* was developed using String online tools (https://string-db.org/) (accessed on 11 March 2023). The results were visualized with Cytoscape v3.9.1.

### 4.4. Plant Tissues and Quantitative Analysis

The plant materials were obtained from Nanjing Forestry University (32°4′ N, 118°48′ E). Young seeds, developing seeds, and mature seeds were collected in August, September, and October 2022, respectively. Female flowers, male flowers, roots, stems, and leaves were collected in July 2022. Three different biological replicates were collected for each tissue. After they were sampled, the tissue samples were placed in liquid nitrogen, frozen, and then stored at −80 °C. We converted the results of the quantitative experiments into data and used Heml 1.0 to visualize the expression results.

## 5. Conclusions

For the first time, this study identified 26 and 20 *GbMADS* genes from two Ginkgo genomes, and they could be divided into types I and II. Compared with *A. thaliana*, Ginkgo had fewer *MADS-box* genes, but its genes had more introns and were longer. The oversized introns of the MIKC genes might be an indicator of higher gene expression. Furthermore, through qPCR, it was found that the type I *GbMADS* genes had a certain effect on the development of male flowers, whereas most of the type II *GbMADS* genes affected the development process of female flowers. In addition, genes of the MIKC subfamily might play important roles in the development of young seeds. Taken together, the results showed that *GbMADS* genes not only had an effect on the sex determination mechanism of Ginkgo but also participated in regulating preproduction. The results of this study are conducive to a better understanding of the structure–function relationship between *MADS-box* gene family members. This study lays an important foundation for exploring the molecular mechanism underlying Ginkgo sex determination.

## Figures and Tables

**Table 1:** Physicochemical properties and intron information of GbMADS.

Gene Name	Sequence ID	Length (aa)	MW (kDa)	pI	GRAVY	Subcellular Localization	Type	Intron
**Version 2019**								
GbMADS01	Gb_31417	381	43,295.54	5.96	−0.672	Nucleus	Mδ	10
GbMADS02	Gb_38365	134	15,267.48	9.34	−0.480	Nucleus	MIKC	2
GbMADS03	Gb_41549	245	28,348.38	9.61	−0.754	Nucleus	MIKC	7
GbMADS04	Gb_41550	201	22,539.02	9.28	−0.051	Nucleus	MIKC	2
GbMADS05	Gb_36364	404	46,738.33	9.32	−0.733	Nucleus	MIKC	7
GbMADS06	Gb_30604	166	19,373.46	10.20	−0.710	Nucleus	MIKC	5
GbMADS07	Gb_38922	165	18,369.30	9.37	0.131	Nucleus	MIKC	1
GbMADS08	Gb_01884	372	42,803.94	9.66	−0.272	Nucleus	MIKC	9
GbMADS09	Gb_19178	246	28,197.98	6.26	−0.594	Nucleus	MIKC	7
GbMADS10	Gb_39109	199	22,576.68	6.22	−0.513	Nucleus	MIKC	5
GbMADS11	Gb_38883	445	50,951.76	5.93	−0.667	Nucleus	Mγ	1
GbMADS12	Gb_28587	257	29,385.11	9.90	−0.416	Nucleus	MIKC	8
GbMADS13	Gb_05359	439	49,658.40	4.70	−0.653	Nucleus	Mγ	3
GbMADS14	Gb_33168	209	24,345.65	5.53	−0.708	Nucleus	Mγ	0
GbMADS15	Gb_12778	91	10,584.23	9.76	−0.693	Nucleus	MIKC	2
GbMADS16	Gb_19258	336	37,202.07	8.33	−0.704	Nucleus	Mα	0
GbMADS17	Gb_03807	190	20,981.41	10.05	−0.104	Nucleus	MIKC	5
GbMADS18	Gb_05128	412	47,476.12	6.33	−0.385	Nucleus	MIKC	9
GbMADS19	Gb_03068	162	18,422.89	9.96	−0.608	Nucleus	MIKC	3
GbMADS20	Gb_16301	380	43,797.09	8.93	−0.226	Nucleus	MIKC	8
GbMADS21	Gb_12586	356	40,514.41	6.32	−0.650	Nucleus	Mα	0
GbMADS22	Gb_21526	169	19,411.53	10.17	−0.499	Nucleus	Mα	0
GbMADS23	Gb_15398	277	31,889.43	8.886	−0.405	Nucleus	MIKC	7
GbMADS24	Gb_40092	336	37,284.17	8.33	−0.680	Nucleus	Mα	0
GbMADS25	Gb_37613	336	37,284.17	8.33	−0.680	Nucleus	Mα	0
GbMADS26	Gb_12581	122	13,740.90	10.02	−0.397	Nucleus	MIKC	2
**Version 2021**								
GbMADS27	GWHPBAVD000173	451	51,348.86	5.61	−0.562	Nucleus	Mδ	12
GbMADS28	GWHPBAVD000308	67	7532.78	10.58	−0.194	Nucleus	MIKC	1
GbMADS29	GWHPBAVD001358	252	29,170.87	8.88	−0.917	Nucleus	MIKC	7
GbMADS30	GWHPBAVD001363	61	6849.08	10.21	−0.292	Nucleus	MIKC	0
GbMADS31	GWHPBAVD001364	245	28,348.38	9.61	−0.754	Nucleus	MIKC	7
GbMADS32	GWHPBAVD001372	61	6881.08	10.29	−0.461	Nucleus	MIKC	0
GbMADS33	GWHPBAVD001859	445	50,951.76	5.93	−0.667	Nucleus	Mγ	1
GbMADS34	GWHPBAVD009827	257	29,385.11	9.9	−0.416	Nucleus	MIKC	8
GbMADS35	GWHPBAVD009828	221	25,387.11	9.73	−0.666	Nucleus	MIKC	6
GbMADS36	GWHPBAVD009829	234	26,706.47	9.26	−0.601	Nucleus	MIKC	7
GbMADS37	GWHPBAVD012282	209	24,345.65	5.53	−0.708	Nucleus	Mγ	0
GbMADS38	GWHPBAVD018550	64	7293.57	10.09	−0.169	Nucleus	MIKC	0
GbMADS39	GWHPBAVD019150	231	26,662.61	9.11	−0.554	Nucleus	MIKC	6
GbMADS40	GWHPBAVD021889	186	21,179.73	9.68	−0.159	Nucleus	MIKC	1
GbMADS41	GWHPBAVD021902	229	26,337	8.96	−0.624	Nucleus	MIKC	7
GbMADS42	GWHPBAVD004734	356	40,514.41	6.32	−0.65	Nucleus	Mα	0
GbMADS43	GWHPBAVD006355	277	31,917.44	8.87	−0.407	Nucleus	MIKC	7
GbMADS44	GWHPBAVD006759	120	13,179.19	9.36	−0.26	Nucleus	MIKC	1
GbMADS45	GWHPBAVD008845	227	26,228.68	6.02	−0.718	Nucleus	MIKC	6
GbMADS46	GWHPBAVD009168	169	19,411.53	10.17	−0.499	Nucleus	Mα	0

## Data Availability

Not applicable.

## Supplementary Materials

The following supporting information can be downloaded at https://www.mdpi.com/article/10.3390/plants12183334/s1. Table S1. Gene ID of more than 80% similarity. Table S2. Primer sequence of GbMADS genes. Table S3. GbMADS genes information homologous to MADS in *Arabidopsis thaliana*. Table S4. Expression of GbMADS genes. Figure S1. Weblogo of *GbMADS* domain. Figure S2. GbMADS domain sequence alignment. S1. GbMADS protein sequences of v2019 genome. S2. GbMADS protein sequences of v2021 genome.
