# Supplementary material for: Identification and Expression of the MADS-box Gene Family in Different Versions of the Ginkgo biloba Genome
Source: Plants (Basel). 2023 Sep 21;12(18):3334. doi: 10.3390/plants12183334 (PMC10535167; doi:10.3390/plants12183334)
Supplement: Supplementary file 1 [file plants-12-03334-s001.zip › plants-2584075-supplementary.pdf]

Supplementary material

Table S1. Gene ID of more than 80% similarity.

Sequence ID of v2019	Sequence ID of v2021	Identity%
Gb_01884 (GbMADS08)	GWHPBAVD001372 (GbMADS32)	90
Gb_12581 (GbMADS26)	GWHPBAVD001363 (GbMADS30)	88.525
Gb_12581 (GbMADS26)	GWHPBAVD001372 (GbMADS32)	88.525
Gb_12586 (GbMADS21)	GWHPBAVD004734 (GbMADS42)	100
Gb_15398 (GbMADS23)	GWHPBAVD006355 (GbMADS43)	99.639
Gb_16301 (GbMADS20)	GWHPBAVD021902 (GbMADS41)	99.103
Gb_21526 (GbMADS22)	GWHPBAVD009168 (GbMADS46)	100
Gb_28587 (GbMADS12)	GWHPBAVD009827 (GbMADS34)	100
Gb_30604 (GbMADS06)	GWHPBAVD018550 (GbMADS38)	81.667
Gb_31417 (GbMADS01)	GWHPBAVD000173 (GbMADS27)	100
Gb_33168 (GbMADS14)	GWHPBAVD012282 (GbMADS37)	100
Gb_36364 (GbMADS05)	GWHPBAVD001358 (GbMADS29)	100
Gb_39109 (GbMADS10)	GWHPBAVD018550 (GbMADS38)	80.328
Gb_41549 (GbMADS03)	GWHPBAVD001364 (GbMADS31)	100
Gb_41550 (GbMADS04)	GWHPBAVD001364 (GbMADS31)	96.667

Table S2. Primer sequence of GbMADS genes.

Gene ID	F	R
GAPDH	GGTGCCAAAAAGGTGGTCAT	CAACAACGAACATGGGAGCAT
GbMADS01	GCAAAATATTGTTCCGCCTAGT	TGTCTTGAGGTGTTGAATGGTG
GbMADS03	CCCTGCCTACGGTGTAAATG	CAATGTGTAGAGTCGGTTCGC
GbMADS04	TCCAGAACCCTGCGTGAAC	CCGATGGTCCCAAGAATGA
GbMADS05	TCGGAAAAAGGAAAGGCTGC	AGGTCCATTATCGTCGGTAGG
GbMADS06	TGGCTGACCTGGAGAGAAAAC	TGCCTCTTTCTCAACCGTATCT
GbMADS07	CAACGCAATGCCACTGAACT	TTCATCATAGACATTTCCCCCA
GbMADS08	ACCACTCCATCATACAAGCCTG	CACGGACTTCAAAAAACTACGG
GbMADS09	ACCAGACTGCAACGTCCATG	CCACTGGCTTGAATCCGAG
GbMADS10	CTCAGAAGTGGAGATTTTGCGT	TTTCTGGTCGGCAGTTTATGTC
GbMADS11	GTCTAGTGCCATCGCAGGAA	GTCTACCAAGGGAGGAGGAAGT
GbMADS12	GAGCGTTCTTTACACAGGCATT	CGAAAAGTTGGTGCTACAGGTG
GbMADS14	AAGAGCAGGGCAAGATGAATAA	TCCGTCTCCAAAAGCCTGTT
GbMADS16	GAAGCGCGAGAAGAAGAGTGA	CGCAGGAGAGAAGACGATAAAA
GbMADS17	CCGTGATTTGGACAGCAGC	CGTGGCAGAAATGACGATGA
GbMADS18	AGTCAGTTGCGACAGAGGGTAA	GCCGAGAAGACGATGAGAGC
GbMADS19	TCCAAATCAAGCGGATAGACA	CCAACTTCGGCATCACAGAG
GbMADS20	AGACACTTGATGGGGGACGC	CGAGAATCAAATCCAGGCAA
GbMADS21	AGCAGTAAAAAAGCACCGAGAG	GGCAAAAGGTTCCCCGAT
GbMADS23	TTTTACAGCCCTTAGAGCCAGC	TCTCACTTTTCACCATTGCCAT
GbMADS26	GTAGGACTGGTTTTATGCGGC	AGACCGAAGAAACAAATGGCT
GbMADS28	ATTCAACGAGCAGGCAGGTC	TGTTTGCCAGTGCTGGAAAA
GbMADS33	TGGGAGATGGCATTGATTTTG	GCAGGGCATCATCCTTAAAGTAT
GbMADS35	AAGCAGTGGTAGCAGCATAAAGC	TGTGTTGAGGTCTTCACCCATC
GbMADS36	CTTTACCAAATGCTTGCTGAACAC	AAGTTTGGTTGGCTTGGCTG
GbMADS39	GCGGCTGTCCGAGTTTGATA	GGTCAGGTCTTCACCCAACAT
GbMADS40	AGAGTCCTCAAGCCTGGTGAA	TCCTCCGCTAGAGAAGACGATA
GbMADS44	CAGGTACGGTCTGGTTGGGT	GGCTGCATCTTCGATTTTCA

Gene ID	F	R
GbMADS45	ATGATGGGTGAACAGATTACGG	GTCGCTCGGTTGTTGGTAAG

Table S3. *GbMADS* genes information homologous to MADS in *Arabidopsis thaliana*.

Query Gene	String Id	Identity	Bitscore
Gb_01884 (GbMADS08)	AGL20	46.2	139
Gb_03068 (GbMADS19)	AGL16	77.3	100.1
Gb_03807 (GbMADS17)	AGL16	63.2	89
Gb_05128 (GbMADS18)	AGL24	40.8	146
Gb_05359 (GbMADS13)	AGL86	31.6	68.6
Gb_12581 (GbMADS26)	AGL6	78	94.7
Gb_12586 (GbMADS21)	AGL61	41.7	109.8
Gb_12778 (GbMADS15)	AGL14	75.3	126.7
Gb_15398 (GbMADS23)	PI	42	172.9
Gb_16301 (GbMADS20)	AG	61.7	266.9
Gb_19178 (GbMADS09)	AGL6	45.6	139.8
Gb_19258 (GbMADS16)	AGL61	36.2	91.7
Gb_21526 (GbMADS22)	AT1G72350	47.5	60.1
Gb_28587 (GbMADS12)	AGL8	45.9	136
Gb_30604 (GbMADS06)	AGL6	49.4	147.5
Gb_31417 (GbMADS01)	AGL104	45.3	180.3
Gb_33168 (GbMADS14)	AGL80	30.8	87
Gb_36364 (GbMADS05)	AGL2	56.2	214.5
Gb_37613 (GbMADS25)	AGL62	39.3	92.8
Gb_38365 (GbMADS02)	AGL6	77.4	96.7
Gb_38883 (GbMADS11)	AT5G26630	37.6	96.7
Gb_38922 (GbMADS07)	AGL6	77.4	98.2
Gb_39109 (GbMADS10)	AGL6	49	142.9
Gb_40092 (GbMADS24)	AGL62	39.3	92.8
Gb_41549 (GbMADS03)	AGL4	52.1	208.8
Gb_41550 (GbMADS04)	3-Sep	85.7	108.2
GWHPBAVD000173 (GbMADS27)	AGL104	45.3	180.3
GWHPBAVD000308 (GbMADS28)	AGL44	85	104.4
GWHPBAVD001358 (GbMADS29)	AGL2	56.2	214.9
GWHPBAVD001363 (GbMADS30)	AGL6	78	93.6
GWHPBAVD001364 (GbMADS31)	AGL4	52.1	208.8
GWHPBAVD001372 (GbMADS32)	AGL6	78	96.3
GWHPBAVD001859 (GbMADS33)	AGL80	31	98.6
GWHPBAVD004734 (GbMADS42)	AGL61	41.7	109.8
GWHPBAVD006355 (GbMADS43)	PI	42	173.3
GWHPBAVD006759 (GbMADS44)	AGL21	69.5	89.7
GWHPBAVD008845 (GbMADS45)	AGL15	44.5	164.1
GWHPBAVD009168 (GbMADS46)	AGL13	42.9	71.2
GWHPBAVD009827 (GbMADS34)	AGL8	45.9	136
GWHPBAVD009828 (GbMADS35)	AP1	48.7	135.2
GWHPBAVD009829 (GbMADS36)	AGL6	42.3	142.9
GWHPBAVD012282 (GbMADS37)	AGL80	30.8	87
GWHPBAVD018550 (GbMADS38)	AGL6	95.1	116.3
GWHPBAVD019150 (GbMADS39)	TT16	53.4	168.7
GWHPBAVD021889 (GbMADS40)	SHP1	72.2	104.4

GWHPBAVD021902 (GbMADS41)	AG	61.7	261.9

Table S4. Expression of GbMADS genes.

	Female Flower	Male Flower	Early Seed	Development Seed	Mature Seed	Root	Stem	Leaf
GbMADS21	122.1	2413.1	79.6	32.1	5.5	17.2	84.4	65.2
GbMADS14	56.8	17198.6	120.4	667.8	226.2	532.5	44.5	377.4
GbMADS11	0.1	81.8	28.5	4.9	31.0	0.1	0.1	0.1
GbMADS33	2.5	52.3	15.8	3.4	32.4	0.1	0.1	0.1
GbMADS16	0.1	2043.3	0.1	0.1	0.1	0.1	0.1	0.1
GbMADS01	0.1	651.1	2.1	15.2	2432.7	0.1	0.1	0.1
GbMADS17	505.5	9.1	10.3	7.7	0.1	0.1	227.3	0.1
GbMADS07	32.3	40.6	56.8	67.3	4.4	0.1	4837.3	6.2
GbMADS08	22047.6	7049.6	4921.9	2015.1	93.4	8.5	14.1	0.1
GbMADS18	4865.4	87.0	2595.3	946.6	80.9	10.9	6848.9	824.1
GbMADS19	8.0	0.1	3.5	6.7	1.6	667.8	44.8	14.5
GbMADS26	191.1	233.9	206.7	219.8	34.5	41.8	0.1	0.1
GbMADS23	272.8	273.1	54.1	27.8	2.3	29.0	48.9	64.1
GbMADS20	40.2	4471.9	17743.5	5634.2	990.3	27.5	21.0	6.6
GbMADS09	713.3	327.8	25.6	13.9	5.3	0.1	0.1	0.1
GbMADS12	49.0	58.9	1.2	3.0	0.1	1.0	0.1	2.7
GbMADS06	8220.4	1964.6	848.2	1045.5	163.3	226.0	328.2	1924.1
GbMADS05	10453.3	6251.6	8769.8	4683.4	217.0	2001.2	98.5	1570.1
GbMADS10	1948.8	643.6	74.8	15.3	264.1	1102.6	1073.7	2309.4
GbMADS03	4765.2	2702.4	1205.2	885.3	352.5	9855.2	0.1	0.1
GbMADS04	26.2	11.5	13.5	11.9	0.1	13.2	0.1	0.1
GbMADS36	39.2	64.1	41.3	63.6	30.8	97.2	158.5	65.8
GbMADS44	52.4	0.1	0.1	0.1	0.1	42.7	65.3	0.1
GbMADS28	20.0	0.1	0.1	0.1	0.1	2452.4	17.6	0.1
GbMADS45	4.3	720.7	1.6	98.4	48.7	78.2	191.6	144.7
GbMADS35	458.8	29.7	3.6	0.1	0.1	0.1	6.9	9.5
GbMADS36	0.1	9280.6	46.7	4.3	2.9	0.1	0.1	0.1
GbMADS39	0.1	90.7	208.7	12707.0	50.4	0.1	0.1	0.1
GbMADS40	5.3	0.1	0.1	0.1	0.1	113.2	0.1	0.1

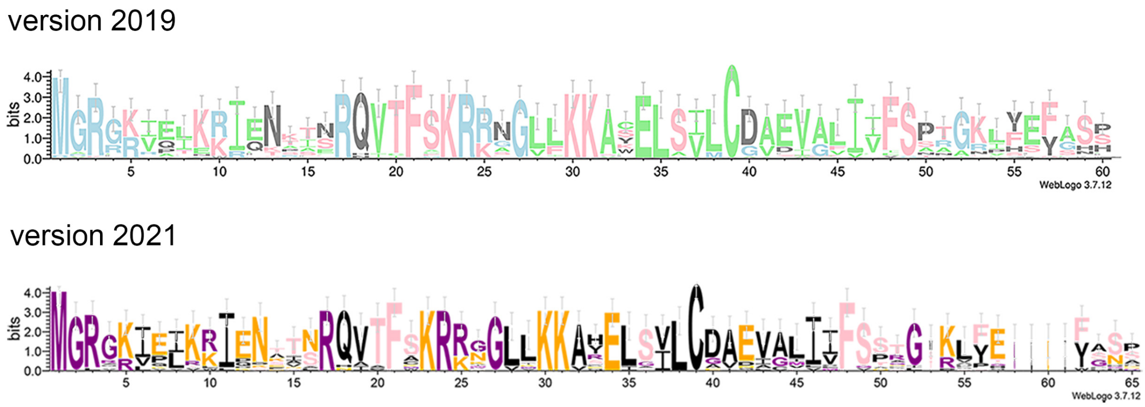

Figure S1. Weblogo of GbMADS domain.

version 2019

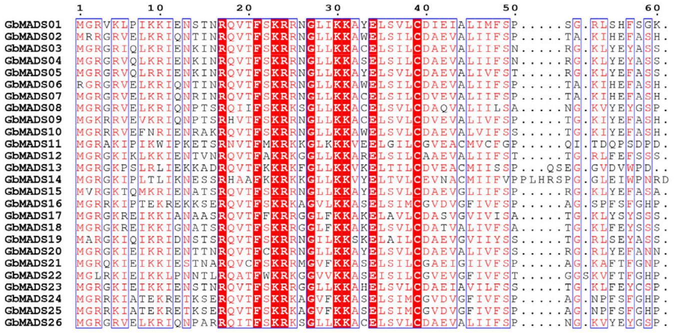

version 2021

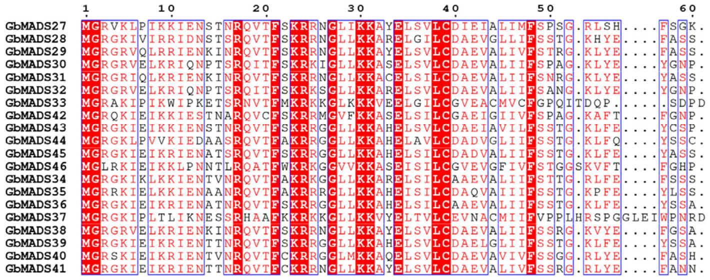

Figure S2. GbMADS domain sequence alignment.

S1. GbMADS protein sequences of v2019 genome.

>GbMADS01
MGRVKLPIKKIENSTNRQVTFSKRRNGLIKKAYELSVLCDIEIALIMFSP
SGRLSHFSGKNSRIEDVIARFVNLPEHERPRLVQNQEYLLRALKKLKYES
DIANHLASPNIVDSNVEELQMDIRRQRIQLEEAQQKLRSFKEDPLLITSI
QDADQYERTLEEALRRVRLRKQQLEHNQMAVAFNDANLQFYIQTQNGLPN
GTDTSQNHLYNSWMPQGDPHTSVQNFMEHENSNAMLAMREAQCMAKCLQN
GTVFPALQDATGMQLPNESASTQPYIPTSHMQFDYTLTDNNNNEHAEQAD
IAAAFDYGSDAMASVHWQTSYGSMTPIVTNQQYPLTKGIMQNIVPPSMSI
YQQDGSSSQGTHHSTPQDNAGMDASFQSNLK
>GbMADS02
MRRGRVELKRIQNTINRQVTFSKRRNGLLKKAWELSILCDAEVALIIFSP
TAKIHEFASHGLYGSPCSEIEIDKIMMMQNPNSPTGETRITNTFQIKWSW
IKESSSPQPDSRHVWNWESTMEPTTMASASVGAT
>GbMADS03
MGRGRIQLKRIENKINRQVTFSKRRNGLLKKACELSILCDAEVALIIFSN
RGKLYEYASSSMSKTLERYQKSLHVIPDTNVTTREAQNWHQEVTKLKGKV
QILQQSQRHLLGEDLGMLSLKELHQLEHQVEVALKHLRSRKTQIMLDQID
DLRKKERMLQEVNKSLHKKFLEADGQNACNFGQFSRPWDSAVGNPAYGVN
EPDSHVQPAHREPTLHIGYREAAHPVSTVHRQKQSANHYTQDWMV
>GbMADS04
MGRGRVQLKRIENKINRQVTFSKRRNGLLKKASELSILCDAEVALIIFSN
RGKLYEYASSRTLREHTLALQSGFYTATDYGDIKTVTGSCVSVCPLCLDS
AVLAHPYCTGQVVLCLECPLPFILGTIGSEYTVQHNIRLLDEDDYDFLES
SILRSLTSKGFSLTNPPLSMVVSLSRGRRLALLRSLNFRSRWAISHRFAD
N
>GbMADS05
DSDKSSSSLHTLSLSASARTTANMKNGLTETADNPPGMNSLHNRSICEPK
CRNRSKSRKRGFEGRTQAIQMGRGRVQLRRIENKINRQVTFSKRRNGLLK
KAYELSVLCDAEVALIVFSTRGKLYEFASSSMNKTLERYEKCSYAVQDTN

VSNREAQNWHQEVTKLKSKVELLQQSQRHLLGEDLGPLSVKELQQLERQL
EIALNHVRSRKSQVMMDLIDELRKKERLLQEVNKSLHKKLSESEGRNATH
DMRHPTDDNGPWNPSVNGGYALPSTQQNTNLHPVDCEPTLQIGYQSVPRE
SIEPPQEQTHNQPQDNYTGWWVNYLISVTSVSQIAHEKSFTTRHVNMPKI
YILSFYNNEAYQYIFFNSVYQNALWPNLASSYHNLWNNLMINNASTFNNF
NSSS
>GbMADS06
MGRGRGRVELKRIQNTINRQVTFSKRRNGLLKKAWELSILCDAEVALIIF
SPTAKIHEFASHGMHNTLCKYKYFSGTTSNYNQDTQNIEYWHIEIERLKA
KMADLERKQKHMIGEDLGSLSSNQLQRLERQLSGGVNKIRLRKRQILSER
IGFLKRKSKICKNNLD
>GbMADS07
MGRGRVELKRIQNRINRQVTFSKRRNGLLKKACELSILCDVEVALIIFSP
TAKIHEFASHGVFIWVWILQRNATELTILLTLSSLFGGNVYDEGENFTPT
RFKALWNWESTMGPTTMASAPASASAGATLELLLFVFGLSLSFCTKESSK
ANEKALAVWALFSNH
>GbMADS08
IHMLGRSHLSKTVGNAILSKEETQQYARMGRGRVELKRIQNPTSRQIIFS
KRKSGLLKKACELSVLCDAQVALIILSANGKVYEYGSPSMNRTLAKYQRF
SSTIDPTTENTKFLRLEAESLHKKMDSLEATLKHMVGENIGSLDLNELKC
LGQHIRVSANKIRKRKRQLFLEDIRKLKTKQRFLQDENAMLNKMVSGVHG
GVQAAEYSMNIMDRVQYQPARDVFTSELPLHHTSLHLGLGQNGNECPQPS
NGINEHGLVTATLLRHVKPNTAVIHNSVNIIYIVRQILSIFYCATHPNFF
EVRECTVIENIHKDNNFADKNHVYNNVFLNVVHVYVNRNCNDHKSCNLNN
KNISIFHKLIVLAKIYNSNRVG
>GbMADS09
MGKRRVEVKRIQNPTSRHVTFSKRRNGLLKKACELSVLCDVEVALIVFSP
TGKIYEFASHSIDETIEKYRTFNEICNDPMDRNCIQSIENRKMTCLEVES
LQKEIDNLQETQKHMIGENLGSLSLEELDKLERKLKVGIDNIYSRKMQIL
SNNCEILVHKVHSLQEENGFLKNMSSNEGHNSVVSPRILSINVMDRLQFQ
PDCNVHDLQSSATFTRQNTSSEASRHPESLQLGFKPVEDASDNYQV
>GbMADS10
MGRRRVEFNRIENRAKRQVTFSKRRNGLLKKAWELSVLCDAEVALVIFSS
TGKIYEFASHGMKETLEKYRNVSTTNEECSPDNQSVEFSCSEVEILRRKR
EALQATQKNMIGEDLGSLSFDDLLQIEGKLQLGANAISSRKEHLLQNKIE
ILQKKLIEQQNSIETSLELSIDINCRPESNVDGYEFPADSIPGKIVVAP
>GbMADS11
MGRAKIPIKWIPKETSRNVTFMKRKKGLKKKVEELGILCGVEACMVCFGP
QITDQPSDPDVWPGMPKALQVIDRYKSLSKEEQDKKKLDNSSFLEQRIKK
LRVELAMKKKENRELEIDTLYPSWDNRLNYFSVEKLRELLDYIDARLAAV
HDRIGFLSRQEQDVENTMQVPLTELESIARQSMEGTAPQMMFPYNLMPCH
ESFTSVSGTVKPYLSLEDQYTTAMCSSSEPNYATAIDYYPSKDHSFVTAL
RDYQTTFKEQLYAVKPIGSSSNNNNNMDQKMVNTDSMAEDRITGVTGYSM
MNKSFPLMGDYEHVQSSAIAGNCSQWRCAVTPLSCCSVHTDSIHNSLYSF
PQHYMNPYGQTANQQTQASCEPQHHQSWLNELIDLQNIQQKLLGDGIDFA
RNNARLPPPLVDRSGTDQDYKLKNLQDLETLQKMKPEYFKDDALQ
>GbMADS12
MYDSSARLSRATSTILILGIIGWVCHIVGDPQQRNMGRGKIKLKKIENTV
NRQVTFAKRKGGLLKKARELSILCAAEVALIIFSTTGRLFEFSSSSMKET
LKRYLCISGKRLWDHQHLISEMATIKKENERLRNALKHVMGNDLNSLSIH
ELQHLEQSLEIAKTRVRTRKNQHILEEMESLRKKERSLHRHYNLLTRILA
RHDGPETVHMTPVAPTFRAQPIQPNLQDIVNQETDLQLGISCAQQTQIPL
NTDLTLS
>GbMADS13
MQPIFILRRHLEGAFEVFSQSQSSKDFLEGGFEVKYSARDNKLCHSPCFA
IRARERFWSPRYEVCSLAAQDKENSNLKQGQSRLDFLPMVCSHGESMGRG
KPSLRLIEKKADRQVTFKKRKFGLLKKVKELTILCDVEACMIISSPQSEG
GVDVWPDMDNAMKVIERYRDLPQEEQGKQKMDNFSLVQQQNEKLENKLKE
ICMQNKHLEMENDYPSWDPHLDNYTIQQLQELTSLINSKMEEAFNSIQSH
KNNIQSMDADNQPVPEAVQTNNEFIPQEQNILSLCPHQDPCPDVQACIDR
LFQQQQQHASTDEPPLPAADNNIVSIDPADANVSFAPVDSSIAPADTNVS
IAPDKTGVDNHMDSAPVDNASADNNVSIAPVDNHMDSAPVDNASNPDFLN

FDPFGIPRSPLFSFTSYESQVFCPKYDFDDSPWNDTIFD
>GbMADS14
MGRGKIPLTLIKNESSRHAAFKKRKKGLLKKVYELTVLCEVNACMIIFVP
PLHRSPGGLEIWPNRDEAMEVITRYRDLPQYEQEQGKMNNLSFLQQQNIN
LDKEFKKKWIQNRLLETENVYPSWDPRLDNYTVEELQDLVSFVNSKMSEV
LDQIQSRENNIQSMDGNQPLPEASNNESLQQNNFSSCTHQGLCPDIEACI
NRLFEEQFP
>GbMADS15
MVRGKTQMKRIENATSRQVTFSKRRNGLLKKAYELSVLCDAEVGLIIFSP
RGKLYEFASASMQKMLERYQKYSDDMNANKKTNEQDAQVIW
>GbMADS16
MGRRKIPTEKREKKSERQVTFSKRKAGVLKKASELSIMCGVDVGFIVFSP
AGSPFSFGHPSVDHLIHKLLRLDPLPPPPAPENLQTHLTQHASLQERYQR
ENVLNKALKLRDNSQPARGQFWWEDDTKNLNPAELKAKYHALKLFKSKLE
LRIAKLRANDGIARPATTDETAGVNNQESDRDRGIEMEAPLLLFETQSKS
NEGEGAATTYDQSCVKQQTTSEPPLVNMTSSCTSAADFIQYQLLHTPPVE
HHNFFQPAMNTDISDQYSSPPGPSSSSWNRTEYGYVGQIPGPAQPQPAFP
PALLPPPPSPLPLLPPPPHIPLPPPPSPPTFANNQN
>GbMADS17
MYSSTVCLTLHLASLVPFLDSYCTEAIWLPRGPVFGEVEYADQYPRHRTL
KPTNGQNNGIDYWAAPSVPPRNNPRAFNRPVIWTAAFQGRIPTTPPNLGG
VLAFKFVDMGRGKREIKKIANAASRQVTFFKRRGGLFKKAKELAVLCDAS
VGVIVISATGKLYSYSSSKVSALVSKLVHSPKILQVIKEQ
>GbMADS18
AVLSRRYGNHDYYICNGLVSHHLPTTAAQHRNRMKSFLGLCMCPAIALIS
SESANLTRILRTVISGSTVMNCESVATEGKRGFSPEKLNNSCDSEEREMG
RGKREIKKIGNATSRQVTFSKRRGGLFKKAKELSVLCDATVALIVFSATG
KLFEYSSSNMKSILERYLKYPEAIERDIPTELIINVDDVELAELNQKYQN
LSLVCRQMSGRDLEGLRFEQLENLEENLEIGLKHVRSRQNELMLMQIDDL
EKGGVQLSEENNNLQLQFDGIFKVGESRPTRDGNTLINFDQLENRDTSSE
CVSTPINMQSNDNAVDEDSEEYETSLQLRLFDPQWRERIRPPNECLRNVS
TTRKMVEKYGFIRTHVNIMRRCLNLDHFKNCQHELISFISNIELLILFTC
AKVDKLLNNFIC
>GbMADS19
METTWFPASDYVHCHKNLRRLPNARSSLQYIINIAASDSEETIHRNDSNE
LIDNRIPVSFLHRGGQRQCKSHDRVGLRMARGKIQIKRIDNSTSRQVTFS
KRRNGILKKSKELAILCDAEVGVIIYSSTGRLSEYASSRSSTVLGEETAS
SKYFGMRLKFTN
>GbMADS20
IHPTVIWISSFFEATIASLSFFLRFAIEDCEDLLNIPTEKMGRGKIEIKR
IENTTNRQVTFCKRRNGLLKKAYELSVLCDAEVALIVFSSRGRLYEFANN
SVKRTIDRYKKTCADNSQGGAISECNSQYWQQEAGKLRQQIDILQNANRH
LMGDALTSLSVKELKQLEIRLERGISRVRSKKNEMLLEEIEIMQRREHIL
LAENQFLRTKIAECESSQNANMLPGPEFDSLPGFDSRHFLHASIMDAHHY
AQQDQTALQLGNSLWAGYITQAQSRFLKMSPRLVCTTYHNNFGATYINEK
TTMELVATSTNMIVISALSISTSRCGNNKTTNCLNFPSKCYLSIENKACF
NPFRPFNFFVSCTITKSYLHFNTSKVNLQL
>GbMADS21
MGRQKIEIKKIESTNARQVCFSKRRMGVFKKASELSILCGAEIGIIVFSP
AGKAFTFGNPCIDYVIDKFLSIPVSLDNEKIQNTVRLERQYNQLLQEQEA
VKKHREQLKRQERNIYNVEREFWWERDISDLDIHQLRQFAAALERLREGI
HNSAEDLQSGNLLPNRSLTAMETLQKPQQSVYYDRESPQVHQIQQTLPSF
PLTSGVQSQLVQWDQESGPNTMGHWNSQEPYNSIGTSFHGPTDAKYSLVQ
FNPAMQDASFVSKRPRLSQHHPALTNIYMASTSTAAGDSSLPSFYEALDL
HKVIISGRQSGFSYPDIAPSDCFMHLSPTVKDNGYDQHEHKNDMASMSES
ARTSEQ
>GbMADS22
MGLRKIEIKKLPNNTLRQATFWKRKGGVVKKASEISILCGVEVGFIVFST
GGSKVFTFGHPSLRHLIHRLHNRPPPPFDNQKLQADLEILEGNYNAFEVR
VKGERAMNTTLKRANKRVQSLCSENKVDFWWAEDTKDFGIAQSKRFYYAL
KLLKTNIQRRMGTSSDVMQ
>GbMADS23

MGLMFLYCFFQFILSIAQWAVEGPLFGFSSIFDHVHCVFLLLSFTALRAS
CGGGKMGRGKIEIKKIENSTNRQVTFSKRRGGLLKKAHELSVLCDAEIAV
ILFSSTGKLFEYCSPRSSIKTVIDRYQRVSGARLWDTQHQNLFSEMAMVK
SENEQLHKTLRHMMGEDVNSLSTDELHSLEQTLEIASSRVRTKKNQYLVQ
QIDKLRKKERFLNEHNNHLYALLVENQASMRDSSTSCQHREQPTAQAFRV
QPSQPNLQDREHDEHHDLRLGFSYFLG
>GbMADS24
MGRRKIATEKRETKSERQVTFSKRKAGVFKKASELSIMCGVDVGFIVFSP
AGNPFSFGHPSVDHLVHKLLRLDPLPPPPAPENFQLHLTQHASLQERYQR
EKVLNKALKLRDNSQPARGQFWWEDDTKNLNPAELKAKYHALKLFKSKLE
LRIAKLRANDGIARPATTDETAGVNNQESDRDRGIEMEAPLLLFETQSKS
NEGEGAATTYDQSCVKQQTTSEPPLVNMTSSCTSAADFIQYQLLHTPPVE
HHNFFQPAMNTDISDQYSSPPGPSSSSWNRTEYGYVGQIPGPAQPQPAFP
PALLPPPPSPLPLLPPPPHIPLPPPPSPPTFANNQN
>GbMADS25
MGRRKIATEKRETKSERQVTFSKRKAGVFKKASELSIMCGVDVGFIVFSP
AGNPFSFGHPSVDHLVHKLLRLDPLPPPPAPENFQLHLTQHASLQERYQR
EKVLNKALKLRDNSQPARGQFWWEDDTKNLNPAELKAKYHALKLFKSKLE
LRIAKLRANDGIARPATTDETAGVNNQESDRDRGIEMEAPLLLFETQSKS
NEGEVGATTYDQSCVKQQTTSEPPLVNMTSSCTSAADFIQYQLLHTPPVE
HHNFFQPAMNTDISDQYSSPPGPSSSSWNRTEYGYVGQIPGPAQPQPAFP
PALLPPPPSPLPLLPPPPHIPLPPPPSPPTFANNQN
>GbMADS26
MGRGRVELKRIQNPARRQITFSKRKSGLLKKACELSVLCDAEVALIIFSP
NGKVYEYGSPRQEELTSGVELDAVWSKGATHVNTLNENSYRSRTGFMRQK
NVYTLISPLRASKKAICFFGLN

S2. GbMADS protein sequences of v2021 genome.

>GbMADS27
MGRVKLPIKKIENSTNRQVTFSKRRNGLIKKAYELSVLCDIEIALIMFSPSGRLSHFSGKNSRIED
VIARFVNLPEHERPRLVQNQEYLLRALKKLKYES
DIANHLASPNIVDSNVEELQMDIRRQRIQLEEAQQKLRSFKEDPLLITSIQDADQYERTLEEALRR
VRLRKQQLEHNQMAVAFNDANLQFYIQTQNGLPN
GTDTSQNHLYNSWMPQGDPHTSVQNFMEHENSNAMLAMREAQCMAKCLQNGTVFPALQDA
TGMQLPNESASTQPYIPTSHMQFDYTLTDNNNNEHAEQAD
IAAAFDYGSDAMASVHWQTSYGSMTPIVTNQQYPLTKGIMQNIVPPSMSIYQQDGSSSQGTHH
STPQDNAGMDASFQNGYVTGQVDLKFLVELIIVMNFG
EDGAISYRKRIIEDFSSMALPLPKERKELDWIAQACEYCVLKYKFFEAGSD
>GbMADS28
MGRGKIVIRRIDNSTSRQVTFSKRRNGLLKKARELGILCDAEVGLLIFSSTGKHYEFASSRFNVL
CS
>GbMADS29
MGRGRVQLRRIENKINRQVTFSKRRNGLLKKAYELSVLCDAEVALIVFSTRGKLYEFASSSMNK
TLERYEKCSYAVQDTNVSNREAQNWHQEVTKLKSKV
ELLQQSQRHLLGEDLGPLSVKELQQLERQLEIALNHVRSRKSQVMMDLIDELRKKERLLQEVN
KSLHKKLSESEGRNATHDMRHPTDDNGPWNPSVNGGY
ALPSTQQNTNLHPVDCEPTLQIGYQSVPRESIEPPQEQTHNQPQDNYTGWWV
>GbMADS30
MGRGRVELKRIQNPTSRQITFSKRKIGLLKKASELSVLCDAEVALIIFSPAGKLYEYGNPR
>GbMADS31
MGRGRIQLKRIENKINRQVTFSKRRNGLLKKACELSILCDAEVALIIFSNRGKLYEYASSSMSKT
LERYQKSLHVIPDTNVTTREAQNWHQEVTKLKGKV
QILQQSQRHLLGEDLGMLSLKELHQLEHQVEVALKHLRSRKTQIMLDQIDDLRKKERMLQEVN
KSLHKKFLEADGQNACNFGQFSRPWDSAVGNPAYGVN
EPDSHVQPAHREPTLHIGYREAAHPVSTVHRQKQSANHYTQDWMV
>GbMADS32
MGRGRVELKRIQNPTSRQITFSKRKSGLLKKARELSVLCDAEVALIIFSANGKLYEYGNPK
>GbMADS33
MGRAKIPIKWIPKETSRNVTFMKRKKGLKKKVEELGILCGVEACMVCFGPQITDQPSDPDVWP
GMPKALQVIDRYKSLSKEEQDKKKLDNSSFLEQRIKK
LRVELAMKKKENRELEIDTLYPSWDNRLNYFSVEKLRELLDYIDARLAAVHDRIGFLSRQEQD

VENTMQVPLTELESIARQSMEGTAPQMMFPYNLMPCH
ESFTSVSGTVKPYLSLEDQYTTAMCSSSEPNYATAIDYYPSKDHSFVTALRDYQTTFKEQLYAV
KPIGSSSNNNNNMDQKMVNTDSMAEDRITGVTGYSM
MNKSFPLMGDYEHVQSSAIAGNCSQWRCAVTPLSCCSVHTDSIHNSLYSFPQHYMNPYGQTAN
QQTQASCEPQHHQSWLNELIDLQNIQQKLLGDGIDFA
RNNARLPPPLVDRSGTDQDYKLKNLQDLETLQKMKPEYFKDDALQ
>GbMADS42
MGRQKIEIKKIESTNARQVCFSKRRMGVFKKASELSILCGAEIGIIVFSPAGKAFTFGNPCIDYVID
KFLSIPVSLDNEKIQNTVRLERQYNQLLQEQEA
VKKHREQLKRQERNIYNVEREFWWERDISDLDIHQLRQFAAALERLREGIHNSAEDLQSGNLLP
NRSLTAMETLQKPQQSVYYDRESPQVHQIQQTLPSF
PLTSGVQSQLVQWDQESGPNTMGHWNSQEPYNSIGTSFHGPTDAKYSLVQFNPAMQDASFVS
KRPRLSQHHPALTNIYMASTSTAAGDSSLPSFYEALDL
HKVIISGRQSGFSYPDIAPSDCFMHLSPTVKDNGYDQHEHKNDMASMSESARTSEQ
>GbMADS43
MGLMFLYCFFQFILSIAQWAVEGPLFGFSSIFDHVHCVFLLLSFTALRASCGGGKMGRGKIEIKK
IENSTNRQVTFSKRRGGLLKKAHELSVLCDAEIAV
ILFSSTGKLFEYCSPRSSIKTVIDRYQRVSGARLWDTQHQNLFSEMAMVKSENEQLHKTLRHM
MGEDVNSLSTDELHSLEQTLEIASSRVRTRKNQYLVQ
QIDKLRKKERFLNEHNNHLYALLVENQASMRDSSTSCQHREQPTAQAFRVQPSQPNLQDREHD
EHHDLRLGFSYFLG
>GbMADS44
MDTKDLESEITPWANIHLTLNSAVKQNKYGTPFNPVRTHTGRYGLVGLIETNSAEDTKIMGRG
KLPVVKIEDAASRQVTFAKRRGGLLKKAHELAVLCDA
DVGLIIFSSTGKLFQYSSCK
>GbMADS45
MGRGKIEIKRIENATSRQVTFSKRRGGLLKKAHELSILCDADVALIIFSSTGKLFEYASSRMKTIL
ERYNKCPEGSQSHLIEYDVEQQYNEVTSLKQQVE
QLQKKQKHMMGEQITDLSVEDLQKLEHQLHEAIHCVRARKDQLVLDQLEEVQKREQHLIEEN
ESLHKQLEEVQKSLPTTERQIAAYLEFQPLESRDFSAK
HVVTRPVAVHNPSFEDSEPSDTSLHLG
>GbMADS46
MGLRKIEIKKLPNNTLRQATFWKRKGGVVKKASEISILCGVEVGFIVFSTGGSKVFTFGHPSLRH
LIHRLHNRPPPPFDNQKLQADLEILEGNYNAFEVR
VKGERAMNTTLKRANKRVQSLCSENKVDFWWAEDTKDFGIAQSKRFYYALKLLKTNIQRRM
GTSSDVMQ
>GbMADS34
MYDSSARLSRATSTILILGIIGWVCHIVGDPQQRNMGRGKIKLKKIENTVNRQVTFAKRKGGLL
KKARELSILCAAEVALIIFSTTGRLFEFSSSSMKET
LKRYLCISGKRLWDHQHLISEMATIKKENERLRNALKHVMGNDLNSLSIHELQHLEQSLEIAKT
RVRTRKNQHILEEMESLRKKERSLHRHYNLLTRILA
RHDGPETVHMTPVAPTFRAQPIQPNLQDIVNQETDLQLGISCAQQTQIPLNTDLTLS
>GbMADS35
MGRRKIELKKIENAANRQVTFAKRRRGLLKKAHEISILCDAQVAIIIFSSTGKPFEYLSSGSSIKQI
LERYQRSTSAWLLDIAHQQLLYDMTRIKNENER
LQNSLRHMMGEDLNTLSTNELHHLEQNLEIATTRVRARKDQLLRQQLEKLTKKLAGHHFPVR
GPVNTYSLYCEGGGPNPLHMTSQHPALAFCPQPSQPNL
QSKEHQQSDLRLGFNCARHQC
>GbMADS36
MGRGKIEIKRIENATNRQVTFSKRRGGLLKKAHELSILCAAEVALIIFSSTGKLFEYSSAGKNLPF
RVFCPYCTSGENIFQHLFCEMTRIKNENERLQTA
MRHMMGEDINSLSTNELHQLEQNLEIAGTRVRTRKNQQMAQQLDKLRKKERFLHQHNNRLY
QMLAEHHEAPMGESSNGYTAFCQQGSNIVHMTPAAPGFR
VQPSQPNLQDNGYQQPDLQLGNDKLSNSFALIIL
>GbMADS37
MGRGKIPLTLIKNESSRHAAFKKRKKGLLKKVYELTVLCEVNACMIIFVPPLHRSPGGLEIWPNR
DEAMEVITRYRDLPQYEQEQGKMNNLSFLQQQNIN
LDKEFKKKWIQNRLLETENVYPSWDPRLDNYTVEELQDLVSFVNSKMSEVLDQIQSRENNIQS
MDGNQPLPEASNNESLQQNNFSSCTHQGLCPDIEACI
NRLFEEQFP
>GbMADS38
MGRGRVELKRIENKINRQVTFSKRRNGLLKKAYELSVLCDAEVALIIFSSRGKVYEFGSAGYLI

>GbMADS39
MGRGKIEIKRIENTTNRQVTFSKRRGGLLKKAHELSVLCDAELGLIIFSSTGKLFEYSSATSSMRK
IIERYQKVSGARLSEFDNQHLFCEMTRIKNENEK
LQTSIRHMLGEDLTSLTMTELHHLEQQLEVAANRVRTRKNQLMLQQLDNLRRKERLLEEQNSH
LCRLLAEHQAAVEGVVAEPMIDFGVFCQSEARNPLHL
TAQSMQGFRLQPTQPNLQESGMQRPALQLWY
>GbMADS40
MTTHTCNYKVNALLDNHDRLSFGKQPHQQGCYVVEKRIKAFEDSRHPPSYPRFSGYKYISLLVI
EIHRVLKPGEAIRPDLIVLRHNRVLCSRPGGLSAES
IRIMGRSKIEIKRIENTVNRQVTFCKRRGGLMKKAQELSVLCDAEVAVIVFSSGGRLYEFASHGY
CPSLQISLYLLVRSCFILQVS
>GbMADS41
MGRGKIEIKRIENTTNRQVTFCKRRNGLLKKAYELSVLCDAEVALIVFSSRGRLYEFANNSVKR
TIDRYKKTCADNSQGGAISECNSQYWQQEAGKLRQQ
IDILQNANRHLMGDALTSLSVKELKQLEIRLERGISRVRSKKNEMLLEEIEIMQRREHILLAENQF
LRTKIAECESSQNANMLPGPEFDALPGFDSRHFL
HASIMDAHHYAQQDQTALQLGSSRPNWLE
